# Distinctive clinical and laboratory features of COVID-19 and H1N1 influenza infections among hospitalized pediatric patients

**DOI:** 10.1007/s12519-021-00432-1

**Published:** 2021-05-10

**Authors:** Ali Alsuheel Asseri, Ayed A. Shati, Saleh M. Al-Qahtani, Ibrahim A. Alzaydani, Ahmed A. Al-Jarie, Mohammed J. Alaliani, Abdelwahid Saeed Ali

**Affiliations:** 1 Department of Child Health, College of Medicine, King Khalid University, Abha, Saudi Arabia; 2 Department of Pediatrics, Abha Maternity and Children Hospital, Abha, Saudi Arabia; 3 General Directorate of Health Affairs, Infection Prevention and Control Administration, Aseer Region, Ministry of Health, Abha, Saudi Arabia; 4 Department of Microbiology and Clinical Parasitology, College of Medicine, King Khalid University, Abha, Saudi Arabia

**Keywords:** Children, Coronavirus disease 2019, H1N1 influenza, Laboratory indices, Symptoms

## Abstract

**Background:**

It had been documented in many studies that pediatric coronavirus disease 2019 (COVID-19) is characterized by low infectivity rates, low mortalities, and benign disease course. On the other hand, influenza type A viruses are recognized to cause severe and fatal infections in children populations worldwide. This study is aimed to compare the clinical and laboratory characteristics of COVID-19 and H1N1 influenza infections.

**Methods:**

A retrospective study comprising 107 children hospitalized at Abha Maternity and Children Hospital, Southern region of Saudi Arabia, with laboratory-confirmed COVID-19 and H1N1 influenza infections was carried out. A complete follow-up for all patients from the hospital admission until discharge or death was made. The clinical data and laboratory parameters for these patients were collected from the medical records of the hospital.

**Results:**

Out of the total enrolled patients, 73 (68.2%) were diagnosed with COVID-19, and 34 (31.8%) were diagnosed with H1N1 influenza. The median age is 12 months for COVID-19 patients and 36 months for influenza patients. A relatively higher number of patients with influenza had a fever and respiratory symptoms than COVID-19 patients. In contrast, gastrointestinal symptoms were observed in a higher number of COVID-19 patients than in influenza patients. A statistically significant increase in white cell counts is noted in COVID-19 but not in influenza patients (*P* < 0.05). There are no obvious variations in the mean period of duration of hospitalization between COVID-19 and influenza patients. However, the total intensive care unit length of stay was longer for influenza compared to COVID-19 patients.

**Conclusions:**

A considerable number of children infected with COVID-19 and H1N1 influenza were noted and reported in this study. There were no significant variations in the severity of the symptomatology and laboratory findings between the two groups of patients. Significant differences between these patients in some hospitalization factors and diagnosis upon admission also were not observed. However, more severe clinical manifestations and serious consequences were observed among pediatric patients hospitalized with influenza infections than among those with COVID-19.

## Introduction

Coronavirus disease 2019 (COVID-19) is a viral infection caused by the novel human coronavirus named severe acute respiratory syndrome coronavirus 2 (SARS-CoV-2) [1–3]. The virus was first reported in late December of 2019 [4]. COVID-19 is a massive epidemic and spread rapidly to cause a global pandemic with high infectivity rates among human populations worldwide. The World Health Organization had declared it as a global public health crisis and pandemic in March 2020 [5]. The clinical manifestations of COVID-19 range from asymptomatic to mild flu-like symptoms, pneumonia, and severe acute respiratory distress syndrome (ARDS) [6, 7]. In children, a peculiar clinical course and epidemiological patterns of COVID-19 were observed and differed from those of adults [8]. The asymptomatic nature of pediatric COVID-19 was observed as the main clinical phenotype and was documented in several epidemiological studies [7, 9, 10]. Mild, moderate, severe, and critical respiratory conditions of pediatric COVID-19 also were observed and reported [11–13]. Fever, dry cough, dyspnea, nasal obstruction, and diarrhea are the suggestive clinical indications of COVID-19 in children [14]. Pneumonia is an uncommon clinical consequence among pediatric COVID-19 patients [11]. Fatal pneumonic cases of pediatric COVID-19 were reported in few cases associated with other comorbidities [10]. Although complications of the disease in children are unlikely to occur, septic shock and ARDS conditions were reported in the critical cases [15–17]. As a general, concrete, and significant conclusion, pediatric COVID-19 was confirmed by low infectivity, morbidity, mortality rates, and benign disease compared to adult cases [8, 18–22].

Influenza was reported as one of the major viral respiratory illnesses of children throughout the globe. It was well-known that influenza viruses A/H1N1 and A/H3N2 are the commonest viruses responsible for acute pediatric influenza infections [23]. It had been reported that the attack rate of influenza among children populations increases in younger and preschool children with a continuous virus shedding [24]. Pediatric influenza is clinically manifested by acute onset of fever, chills, runny nose, cough, sore throat, headache, and myalgia [25, 26]. Digestive disorders like diarrhea and vomiting are not frequent to occur among pediatric influenza cases [27]. Pneumonia and bronchiolitis are the frequently occurring complications observed in infants and young children infected with influenza [24, 27]. Conclusively, influenza is a common and fatal viral respiratory infection in children of all ages worldwide [28, 29]. Although the comparison of the clinical and epidemiological features of COVID-19 and influenza had been made in some similar previous studies [28, 30], in this communication, we report for the first time a comparative evaluation of the clinical and laboratory characteristics of COVID-19 and influenza infections among children as inpatients. We assume that understanding these two diseases' distinctive and characteristic features among pediatric patients will help in the early diagnosis, treatment, and preventive measures that have to be implemented.

## Methods

### Study population and setting

This retrospective study included children diagnosed with laboratory-confirmed COVID-19 and H1N1 influenza infections and hospitalized between April 15 and November 20, 2020, for COVID-19 and from August 2019 to March 2020 for H1N1 influenza at Abha Maternity and Children Hospital (AMCH). AMCH is a referral, tertiary care, and teaching hospital at Abha, the southwestern region's capital, Saudi Arabia. The study included pediatric patients admitted in both the general pediatric wards and the pediatric intensive care units (PICU). A confirmed case of both COVID-19 and influenza is given positive results using the reverse-transcriptase polymerase chain reaction test in a nasopharyngeal swab specimen. The selected children are those who confirmed positive for either of the two diseases during the hospitalization period. Those who tested positive for the viruses at the preadmission phase were excluded. Children diagnosed with any other respiratory infections, such as respiratory syncytial virus, parainfluenza, and rhinovirus, were also excluded. One hundred and seven children were selected and included in the final evaluation; 73 children were confirmed COVID-19 patients, and 34 confirmed influenza patients. The institutional review board at AMCH approved the study.

### Data collection

The demographic data, clinical characteristics, symptoms at presentation, and comorbid conditions were collected from the AMCH medical records. All the patients' laboratory results, medical interventions, duration of hospitalization, diagnosis at admission, oxygen therapy, and outcomes were also collected.

### Statistical analysis

Statistical analyses were performed using Stata version 14 (StataCorp, College Station, Texas). Parametric test (Student’s *t* test) and non-parametric test [two-sample Wilcoxon-rank-sum (Mann–Whitney *U* test)] were used to study the differences between normally and non-normally distributed continuous variables, respectively. Fisher exact was used to study the differences between categorical variables. Categorical and continuous data were presented as proportions and median with inter-quartile range, respectively. A *P* value of < 0.05 was used to determine the statistical significance.

## Results

### Baseline demographics and clinical characteristics of pediatric COVID-19 and H1N1 influenza infections

The demographic data and clinical characteristics of COVID-19 and H1N1 influenza-infected children are summarized in Table 1. One hundred and seven children hospitalized with the two diseases are included in the study. Out of these patients, 73 (68.2%) were diagnosed with COVID-19, and 34 (31.8%) were diagnosed with H1N1 influenza. 54.8% of these children were males, whereas 45.2% were females. Significant variations (*P* < 0.05) between the two groups of patients were observed based on previous contact history with infected individuals. 89.0% of COVID-19 patients had a history of contacts, while 70.6% of influenza patients were reported had previous contacts with infected individuals. As for the presenting symptoms, significant differences (*P* < 0.05) between the two groups of patients were noted for the cough, shortness of breath (SOB), runny nose, and diarrhea. Cough, SOB, and runny nose were detected in a higher number of influenza than COVID-19 patients. In contrast, diarrhea was detected in about 27.4% of COVID-19 patients, and no case of influenza patients presented with diarrhea. There were no significant differences between the two groups of patients observed for fever, convulsions, and vomiting.

**Table 1:** Demographic and clinical characteristics of children diagnosed with COVID-19 and H1N1 influenza

Variables	COVID-19 (*n* = 73)^a^	H1N1 Influenza (*n* = 34)^a^	Total (*n* = 107)^a^	*P*
Age (mon), median (IQR)	12 (2–60)	36 (12–72)	24 (6–72)	0.05^*^
Male, *n* (%) Female, *n* (%)	40 (54.8)^b^ 33 (45.2)	19 (55.9) 15 (44.1)	59 (55.1) 48 (44.8)	0.54
History of contact, *n* (%)	65 (89.0)	24 (70.6)	89 (83.2)	0.02^*^
Initial ER symptoms, *n* (%)				
Fever	70 (95.9)	33 (97.0)	103 (96.3)	0.62
Cough	42 (57.5)	33 (97.0)	75 (70.1)	< 0.001^*^
Shortness of breath	33 (45.2)	29 (85.3)	62 (57.9)	< 0.001^*^
Runny nose	23 (31.5)	29 (85.3)	52 (48.6)	< 0.001^*^
Convulsion	5 (6.8)	2 (5.9)	7 (6.5)	0.61
Vomiting	24 (32.9)	6 (17.6)	30 (28.0)	0.07
Diarrhea	20 (27.4)	0 (0.0)	20 (18.7)	< 0.001^*^
Previously healthy	46 (63.0)	17 (50.0)	63 (58.9)	0.14
Comorbid conditions				
Asthma, *n* (%)	10 (13.7)	8 (23.5)	18 (16.8)	0.16
CHD, *n* (%)	8 (10.9)	3 (8.8)	11 (10.3)	0.51
Epilepsy, *n* (%)	5 (6.8)	8 (23.5)	13 (12.1)	0.01^*^
Genetic disorder, *n* (%)	5 (6.8)	4 (11.8)	9 (8.4)	0.30
Hematological disorders, *n* (%)	3 (4.1)	2 (5.9)	5 (4.7)	0.51
Rheumatological disorders, *n* (%)	6 (8.2)	1 (2.9)	7 (6.5)	0.28
Respiratory insufficiency, *n* (%)	17 (23.3)	7 (20.6)	24 (22.4)	0.48
Prematurity, *n* (%)	14 (19.2)	2 (5.9)	16 (15.0)	0.06
Home oxygen use, *n* (%)	5 (6.8)	5 (14.7)	10 (9.3)	0.17
Temperature, *n* (%)	38.0 (37.5–38.4)	37.6 (37.0–38.3)	38.0 (37.0–38.4)	0.13
SaO_2_ level (%), median (IQR)	94 (88–96)	87 (83–90)	90 (85–95)	< 0.001^*^
Respiratory rate (breaths/min), median (IQR)	32 (28–44)	34 (30–45)	32 (28–45)	0.36

*P* values were calculated using two-sample Wilcoxon-rank-sum for the non-normally distributed continuous data. Fisher exact was used to study the differences between categorical variables. *COVID-19* coronavirus disease 2019, *IQR* Interquartile range, *ER* emergency room, *CHD* congenital heart disease, *SaO*_*2*_ arterial oxygen saturation. ^a^Number of patients from each group of children; ^b^Number of children for each respective variable (%). ^*^P < 0.05 statistically significant

Regarding the comorbidities, epilepsy differs significantly between the two infections; 23.5% of influenza and 6.8% of COVID-19 patients had had epilepsy (*P* < 0.05). Other comorbid conditions did not differ significantly between COVID-19 and influenza patients.

### Laboratory results of COVID-19 and influenza among pediatric patients

Table 2 summarizes the initial laboratory results for the two groups of patients. Significantly higher levels and increase in the total white cells, absolute neutrophils, absolute lymphocytes, and platelet counts were observed among COVID-19 than influenza patients (*P* < 0.05 for all). No statistical significance was observed for the hemoglobin, C-reactive protein, albumin levels, and erythrocytes sedimentation rates between the two groups of patients. As for the liver enzymes analysis, significant differences between the two groups of patients regarding alanine aminotransferase (ALT) and aspartate aminotransferase (AST) were reported. Higher levels of both ALT and AST were observed in influenza than COVID-19 patients.

**Table 2:** Initial laboratory findings and hematological predictors for pediatric COVID-19 and H1N1 influenza patients

Variables	COVID-19 (*n* = 73)	Influenza H1N1 (*n* = 34)	Total (*n* = 107)	*P*
White blood cell count (× 10^3^/μL, ref. 4.3–11.0 × 10^3^/μL), median (IQR)	9.3 (6.2–14.0)	5.6 (2.8–8.6)	7.4 (5.2–13.0)	< 0.001^*^
Neutrophil count (absolute neutrophil count, ref. 1500–8500 cells/µL), median (IQR)	3894 (2100–7340)	1930 (960–4970)	2953 (1380–6580)	0.002^*^
Absolute lymphocyte count (cells/μL, ref. 970–3960/μL), median (IQR)	3360 (1848–5085)	1850 (960–3472)	2810 (1470–4760)	0.005^*^
Hemoglobin level (g/dL, ref. 11.5–15.5 g/dL), mean ± SD	11.5 ± 2.0	12.4 ± 2.0	11.7 ± 2.0	0.20
C-reactive protein (mg/dL, ref. 0.0–0.9 mg/dL), median (IQR)	0.8 (0.0–1.6)	0.8 (0.0–1.6)	0.8 (0.0–1.6)	0.81
Erythrocyte sedimentation rate (mm/h, ref. 0–15 mm/h), median (IQR)	15 (7–34)	18 (12–40)	17 (10–35)	0.22
Platelets (× 10^3^/μL, ref. 150–400 × 10^3^/μL), mean ± SD	316 ± 156	256 ± 119	297 ± 147	0.05^*^
Alanine aminotransferase (U/L, ref. 10–35 U/L), median (IQR)	20 (14–26)	29 (21–38)	22 (17–30)	0.002^*^
Aspartate aminotransferase (U/L, ref. 10–34 U/L), median (IQR)^a^	23 (18–33)	56 (37–97)	28 (19–48)	< 0.001^*^
Albumin (g/dL, ref. 3.7–5.6 g/dL), median (IQR)	3.4 (2.8–3.7)	3.5 (3.1–3.6)	3.4 (2.8–3.7)	0.60

*P* values were calculated using Student’s *t* test and two-sample Wilcoxon-rank-sum to study the differences between normally and non-normally distributed continuous variables, respectively. *COVID-19* coronavirus disease 2019, *IQR* interquartile range of laboratory values, *SD* standard deviation. ^a^There were three missing values for aspartate aminotransferase and alanine aminotransferase in the influenza H1N1 group. ^*^P < 0.05 statistically significant

### Hospitalization factors and admission diagnosis

The hospitalization factors and diagnosis upon admission for both pediatric COVID-19 and influenza patients are shown in Tables 3 and 4, respectively. There were no significant differences in the total and general ward duration of hospitalization between the two groups of patients. However, a significant difference and more days of PICU hospitalization for influenza patients were observed compared to COVID-19 patients. A significant difference (*P* < 0.05) between the two groups of patients in their need for oxygen therapy also was observed. More days of oxygen therapy were required for COVID-19 than for influenza patients.

**Table 3:** Comparative evaluation of pediatric COVID-19 and H1N1 influenza for some hospitalization factors

Hospitalization factors	COVID-19 (*n* = 73)	H1N1 influenza (*n* = 34)	*P*
Overall hospitalization duration (d)	6 (3–10)^a^	5 (4–7)	0.80
In general pediatric ward (d)	4 (3–7)	5 (4–5)	0.40
In PICU (d)	4 (2–7)	9 (8–25)	0.01^*^
Oxygen therapy (d)	6 (5–14)^a^	4 (3–7)	0.03^*^

Data are median (IQR). *P* values were calculated using two-sample Wilcoxon-rank-sum, because data were not normally distributed. *COVID-19* coronavirus disease 2019, *IQR* interquartile range of laboratory values, *PICU* pediatric intensive care unit. ^a^Median number of days (the interquartile range of days for hospitalization and oxygen therapy periods). ^*^P < 0.05 statistically significant

**Table 4:** H1N1 influenza and COVID-19 infected children stratified by the admission diagnosis

Clinical diagnosis	COVID-19 (*n* = 73)	H1N1 (*n* = 34)	*P*
Acute pneumonia	15 (20.5)	13 (38.2)	0.046^*^
Acute bronchiolitis	7 (9.6)	12 (35.3)	0.001^*^
Acute gastroenteritis	15 (20.5)	0 (0.0)	0.002^*^
Clinical sepsis	12 (16.4)	0 (0.0)	0.007^*^
Asthma exacerbation	4 (5.5)	4 (11.8)	0.22
Acute meningitis	2 (2.7)	0 (0.0)	0.33
Complicated pneumonia	2 (2.7)	3 (8.8)	0.16

Data are *n* (%). *P* values were calculated using Fisher exact was used to study the differences between categorical variables. *COVID-19* coronavirus disease 2019. ^*^P < 0.05 statistically significant

Significant differences (*P* < 0.05) between the two groups of patients were observed regarding the diagnosis upon admission for acute pneumonia, acute bronchiolitis, acute gastroenteritis, and clinical sepsis. Diagnosis of acute pneumonia and bronchiolitis was given to more patients confirmed with influenza than those with COVID-19 infection. While the diagnosis of acute gastroenteritis and clinical sepsis was given only to COVID-19 patients, these diagnoses were not given to any of the influenza patients. There were no significant variations observed between the two groups of patients in the number of children diagnosed with asthma exacerbation, acute meningitis, and complicated pneumonia upon admission.

## Discussion

Viral respiratory infections (VRIs) constitute major infectious threats among children populations worldwide. Acute VRIs represent the primary cause of deaths among children in developing countries [31]. Influenza and influenza-like infections are common among pediatric patients and seem responsible for hospitalization in many parts of the world. To our knowledge, this is the first study to compare the clinical and laboratory characteristics of influenza and COVID-19 infections among hospitalized pediatric patients in Saudi Arabia and the Middle East at large. In the present study, we aimed to compare the clinical and laboratory features between the newly identified COVID-19 and A/H1N1 influenza infections among the pediatric patients hospitalized in AMCH, Southern region of Saudi Arabia, both in the general pediatric units and PICU. Although influenza had been reported in several previous studies to constitute the major cause of hospitalization of children of all ages [32–34], our study revealed a different picture. That is acceptable at this point as the COVID-19 pandemic impacted the human populations globally with heavy health burdens.

A relatively higher number of patients who had previous contacts with infected individuals among the pediatric COVID-19 patients than among the influenza patients is reported in this study. This is an acceptable finding in the light of the heightened awareness of COVID-19, compared to influenza, among the communities, while the disease pandemic impacts the human populations throughout the world. On the contrary, the recommended annual vaccination against influenza infections for different individuals largely reduced the risk of infections requiring hospitalization, ICU admission, and death [35]. On another note and in a recent unprecedented study conducted by Akin and Gözel (2020) to understand the COVID-19 dynamics in contrast to influenza, they concluded that COVID-19 spread and transmissibility rates are similar to the Spanish flu (in 1918), Asian flu (in 1957), Hong Kong flu (in 1968), and swine flu (in 2009) when they emerged [36]. Despite all these data, we believe that our study findings cannot be considered strong conclusive remarks to compare the transmissibility between the COVID-19 and influenza among the children populations due to the limited number of patients tested and the biased region of study and hospital selection.

The results obtained in this study also showed that significant differences (*P* < 0.05) between the two groups of patients were observed in manifesting respiratory symptoms. The results indicated that symptoms, including dry cough, SOB, and runny nose, were observed in a higher number of influenza patients than COVID-19. These findings substantiate the well-established and documented idea that COVID-19 is an asymptomatic or mild disease in children, and only a few severe cases associated with comorbidities can be encountered [7, 8, 11]. In a similar previous study, we intensively reviewed and reported several explanations for this benign COVID-19 among pediatric patients from many scientific and cultural perspectives [8]. In addition, a recent study had also shown that children vaccinated against diphtheria, tetanus, and pertussis infections (using the DTP vaccine) might be protected against severe COVID-19 infections, as DTP vaccines had been suggested to have significant sources of potential cross-reactive immunity to SARS-CoV-2 [37].

On the contrary, influenza was associated with severe presenting respiratory symptoms in children [38]. Furthermore, more than one-quarter (27.4%) of the confirmed COVID-19 patients in this study presented with diarrhea, whereas diarrhea was not reported among those diagnosed with influenza. This can serve as a potential distinctive tool and differential diagnostic feature between the two infections in children. Relatively high COVID-19 patients presented with diarrhea in this study were reported compared to the previous similar studies. The association of diarrhea with COVID-19 had been extensively discussed and justified in many previous reports [39–41]. Concerning the comorbidities, in both groups of patients, comorbidities including asthma, congenital heart disease, genetic disorders, hematological disorders, rheumatologic disorders, respiratory insufficiency, and prematurity were reported without significant differences. These were preexisting conditions, and thus both types of patients are expected to be presented with them. These conditions should play some roles in complicating viral infections in this context. Severe and fatal pediatric COVID-19 cases were observed if accompanied by preexisting comorbidities [42]. However, lacking the statistical significance in our data is most probably due to the small sample size.

As for the laboratory indices, the total and absolute white blood cells and platelet counts were observed higher among COVID-19 than influenza patients. Similar results were also obtained in several previous studies [43, 44]. However, leucopenia and lymphopenia were recognized as associated with severe COVID-19 infections [28, 44]. It was also reported that liver injury occurs during some severe cases of COVID-19 resulting in abnormally higher liver enzyme levels [6, 45]. This justifies our findings in this report, which declare a slight increase in the liver enzymes (ALT and AST) among the COVID-19 patients. On the other hand, the increase in the liver enzymes associated with influenza infections was discussed in a few previous reports [46]. Despite all of that, these liver enzymes showed significantly higher levels among influenza patients than COVID-19 in this scientific communication.

When using some hospitalization factors to compare between the pediatric COVID-19 and influenza cases in this study, it can obviously be noticed that more days of stay in the PICU are required for influenza than COVID-19 patients. However, this situation depends on the severity of the disease in both cases. This indicated that influenza might result in more severe and serious clinical consequences in children as compared with COVID-19, which confirmed had benign clinical features among children populations in many parts of the world [8]. However, the required period of oxygen therapy among these hospitalized patients was seen longer for COVID-19 cases than influenza cases. Oxygen therapy is required for critically ill conditions in most types of respiratory infections. It had been confirmed in several studies that prolonged mechanical ventilation is required for COVID-19 management to relieve the ARDS, which is more likely to supervene in severe cases [47, 48]. In addition, it had also reported that influenza patients are more responsive to oxygenation than COVID-19 patients [49]. This justifies the more days used for oxygen therapy for COVID-19 patients than influenza, as shown in our study findings. Our findings in this study indicated that some severe COVID-19 cases might have been hospitalized during the study phase.

In contrast to COVID-19 cases among hospitalized patients in this study, significantly higher proportions of influenza patients were primarily diagnosed with acute pneumonia and acute bronchiolitis. This may be attributed to the more likely occurring bacterial co-infection, causing bronchopneumonia among influenza patients who are relatively older than COVID-19 infected children. The low rates of bacterial co-infections associated with COVID-19 patients had previously been reported [49, 50]. It is a much acceptable and logical idea having given the preadmission diagnosis of gastroenteritis to COVID-19 patients as it had been published previously that the causative virus of the disease infects the epithelial cells of the small intestine in humans, where they abundantly express the angiotensin converting enzyme 2 receptors of the virus [40, 51]. In the present study, some conditions accompanied the infection in the COVID-19 patients, which were not seen among the influenza patients.

Similarly, COVID-19 was also observed associated with many conditions in children during the early days of the pandemic. These conditions, such as toxic shock syndrome and Kawasaki-like syndrome, were collectively termed by the Centers for Disease Control as multisystem inflammatory syndrome in children (MIS-C) [52]. Unlike many previous reports in the literature that showed that pediatric COVID-19 was accompanied by a dearth of cases of MIS-C, our findings in this study revealed a considerable number (11%) of COVID-19 patients diagnosed with MIS-C. On the contrary, it had also reported that influenza was not seen associated with MIS-C [53]. Again, this suggests that COVID-19 may manifest more severe clinical symptomatology and pathology among pediatric patients than influenza. It is also suggestive of more poor prognostic disease conditions among COVID-19 patients than influenza patients. The considerably higher MIS-C cases in this study are attributed to the fact that our data were collected from a tertiary and referral hospital in the study area. Therefore, those presented and admitted to the hospital are mostly severely sick patients. This is beside the availability of most of the pediatric subspecialties who diagnosed and managed such complicated diseases. In addition, the global awareness about this newly described condition in children (MIS-C) is another substantial reason for having a relatively higher number of MIS-C cases associated with COVID-19.

This study's limitations included the small sample size, which may have limited the ability of our analysis to detect small differences between the two groups in certain variables. The retrospective nature of the study may also serve as a limiting factor given the chance of bias. In addition, some disease severity factors, such as the mortalities, nutritional status, socioeconomic status, and management, were not used as comparative elements between patients.

In conclusion, although there were no significant differences in respiratory symptoms and many laboratory correlates and hospitalization factors noted between the COVID-19 and H1N1 influenza infections among hospitalized pediatric patients, influenza may produce more critical disease with more severe clinical phenotypes as compared to COVID-19.
